# Towards Quality Assessment for Arbitrary Translational 6DoF Video: Subjective Quality Database and Objective Assessment Metric

**DOI:** 10.3390/e27010044

**Published:** 2025-01-07

**Authors:** Chongchong Jin, Yeyao Chen

**Affiliations:** 1 College of Science and Technology, Ningbo University, Ningbo 315300, China; 2 Faculty of Information Science and Engineering, Ningbo University, Ningbo 315211, China; chenyeyao@nbu.edu.cn

**Keywords:** arbitrary translational 6DoF, synthesized video, subjective and objective, video quality assessment

## Abstract

Arbitrary translational Six Degrees of Freedom (6DoF) video represents a transitional stage towards immersive terminal videos, allowing users to freely switch viewpoints for a 3D scene experience. However, the increased freedom of movement introduces new distortions that significantly impact human visual perception quality. Therefore, it is crucial to explore quality assessment (QA) to validate its application feasibility. In this study, we conduct subjective and objective QAs of arbitrary translational 6DoF videos. Subjectively, we establish an arbitrary translational 6DoF synthesized video quality database, specifically exploring path navigation in 3D space, which has often been limited to planar navigation in previous studies. We simulate path navigation distortion, rendering distortion, and compression distortion to create a subjective QA database. Objectively, based on the spatio-temporal distribution characteristics of various distortions, we propose a no-reference video quality assessment (VQA) metric for arbitrary translational 6DoF videos. The experimental results on the established subjective dataset fully demonstrate the effectiveness and superiority of the proposed objective method.

## 1. Introduction

**Figure 1:**
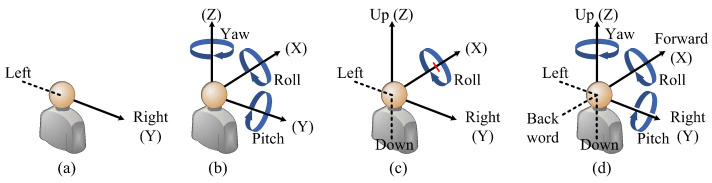
Illustration of DoF scopes regarding different types of immersive videos. (**a**) Free viewpoint video (FVV) with translational 1DoF. (**b**) Panoramic video with rotational 3DoF. (**c**) Windowed 6DoF video with translational 2DoF and rotational 1DoF. (**d**) 6DoF video with arbitrary translational 3DoF and rotational 3DoF.

As an emerging media that can experience richer sensory stimuli with the naked eye, immersive video has been gradually applied to many fields such as scene imaging [1,2], multi-view clustering [3], and medical examination [4]. The Moving Picture Experts Group (MPEG) defines immersive video as different stages according to the evolution of the Degree of Freedom (DoF). The DoF refers to the minimum coordinate dimension required to determine the spatial position of a target. Figure 1a–d illustrate the DoFs when users navigate the Free Viewpoint Video (FVV) [5], panoramic video [6], windowed 6DoF video, and 6DoF video [7], respectively. Compared with 1DoF in the FVV system, 3DoF in the panoramic video system, and restricted 6DoF in the windowed 6DoF video system, 6DoF includes three translational DoFs and three rotational DoFs, with which the users can freely appreciate the scene. Hence, 6DoF is the ultimate goal of immersive video.

By briefly summarizing the above video systems with different DoFs, we can find that the scope of freedom of video can be roughly divided into two aspects: translational DoFs (moving left–right, up–down and forwards–backward) and rotational DoFs (pitch, yaw, and roll). Whenever there is a need to achieve translational DoFs, this requires rendering between viewpoints, which involves the use of a small number of original viewpoints to generate virtual viewpoints to fill the gaps between viewpoints and reproduce the occluded scenes, so that the viewpoint switching is more coherent and meets the users’ comfortable visual experience. Depth Image-Based Rendering (DIBR) technology is a common method for generating virtual viewpoints [8]. However, rendering introduces new types of distortions that are significantly different from traditional distortions such as blurring and noise [9]. For example, rendering distortions are often distributed around object edges, disrupting the geometric structure of the scene, while traditional distortions are typically distributed globally, compromising the clarity of the scene. As the DoF of immersive video increases, it becomes necessary to render content in various interactive dimensions, including rendering between left–right, up–down, and front–back viewpoints, and their mixed conditions. Under these circumstances, the distortions generated by the video become very complex, significantly affecting the user’s perception and experience. Therefore, it is necessary to conduct a quality assessment (QA) on the synthesized video to determine whether its quality meets the user’s needs.

Currently, the QA of immersive videos related to rotational DoFs, such as panoramic videos, has become increasingly mature. However, the research on QA for immersive videos involving translational DoF is still at the FVV stage (i.e., translational 1DoF stage). To address this issue, Jin et al. explored the QA of windowed 6DoF videos [10], upgrading 1DoF to 2DoF. However, such videos still cannot support viewpoint switching forward and backward, and the research on translational DoF is still inadequate. To better approximate the quality estimation of 6DoF videos, this paper conducts subjective and objective QA studies for 6DoF videos with translational DoFs (temporarily abbreviated as arbitrary translational 6DoF). The main contributions are as follows:

(1) This paper establishes a new arbitrary translational 6DoF synthesized video quality database for exploring higher-DoF video quality. The database contains five sequences, four levels of compression distortion, and four levels of rendering distortion, with particular focus on three viewpoint navigation paths in 3D space that have not been explored in previous research.
(2) For the specific distortion types of arbitrary translational 6DoF synthesized videos, this paper proposes a no-reference objective quality assessment method. The proposed method leverages multiscale and multi-resolution statistical modeling to extract features for cracks, rendering, and compression distortions, thereby achieving an effective description of distortions with complex spatio-temporal and local–global distributions.
(3) The established subjective QA database offers a novel aspect of view path navigation that previous databases lack. Meanwhile, the proposed objective QA method leverages the spatio-temporal characteristics of distortions to explore the impact of high-DoF distortions on video quality. Extensive experimental results on the established subjective dataset fully validate the superiority of the proposed objective method.

The rest of the paper is organized as follows. Section 2 briefly summarizes the relevant works of subjective and objective research in the field of synthesized video. Section 3 introduces the subjective study of establishing exploratory arbitrary translational 6DoF synthesized video quality database. In Section 4, an objective arbitrary translational 6DoF synthesized QA metric is developed. The experimental results are discussed in Section 5. The conclusion is given in Section 6.

## 2. Related Works

### 2.1. Subjective Quality Assessment

Currently, the FVV supports a 1DoF with transitional viewpoint switching [11]. The synthesized video in the FVV system suffers geometric distortions, e.g., holes and artifacts. The subjective QA of FVV has been extensively studied, and two subjective QA databases, the IRCCyN/IVC DIBR database [12] and the Shenzhen Institute of Advanced Technology (SIAT) database [13], are presented. The IRCCyN/IVC DIBR video quality database includes 102 video samples which involve three sequences, rendering, and compression distortions. The subjective score, named the Mean Opinion Score (MOS), was assigned by humans through the Absolute Category Rating with Hidden Reference (ACR-HR) [14]. The SIAT database includes 140 video samples generated based on ten sequences. Rendering distortion on the View Synthesis Reference Software platform (VSRS-1D-Fast) [15] and compression distortion are introduced. The above two common databases mainly study the quality degradation caused by rendering distortion and compression distortion in translational 1DoF.

In order to explore the subjective perceptual quality differences caused by different viewpoint switching paths, Ling et al. [16] further considered the impact of different viewpoint navigation scan paths on the video quality while considering rendering and encoding processing, and established an IPI-FVV database. Furthermore, Yan et al. [17] studied the problem of viewpoint switching from annular arrangement sources and established a rendered video database called the Youku-FVV database. Although these two datasets simulate the impact of different paths on the subjective perceptual quality, regardless of the increase in rotational DoF, their viewpoint switching still remains at the level of translational 1DoF. It has not yet achieved a high-dimensional breakthrough in mobility.

To address the limitation of translational 1DoF, Jin et al. built a windowed 6DoF synthesized video database with translational 2DoF [10], which includes four compression states, four rendering schemes, and two view switching trajectories. As a result, a total of 128 synthesized videos were generated. Compared with the above two FVV databases, the videos in this windowed 6DoF database are synthesized along both the horizontal and vertical dimensions, and achieve translational DoF in both the left–right and up–down directions. Unfortunately, the synthesized videos in [8] still cannot achieve forward and backward viewpoint switching, limiting the true DoF of movement. Therefore, in order to solve this problem, we plan to further add forward- and backward-moving DoFs to the windowed 6DoF database and establish a new synthesized video database. Note that, due to the simultaneous implementation of left–right, up–down, and forward–backward movement in this database, we named it the arbitrary translational 6DoF synthesized video database.

### 2.2. Objective Quality Assessment

Due to the subjectivity of viewpoint switching, the proposed database does not contain reference videos. Therefore, we mainly summarize the existing no-reference (NR) objective quality evaluation methods, including Image QA (IQA) and Video QA (VQA).

(1) NR traditional IQA/VQA: There are some classic NR IQA and VQA metrics for traditional 2D images/videos. For example, Moorthy et al. [18] proposed a general Blind Image Quality Index (BIQI), which utilizes multidimensional statistical features extracted in the discrete wavelet domain to measure the image quality. Liu et al. [19] built a Spatial Spectral Entropy-based quality index (SSEQ), which employs spatial entropy and spectral entropy to assess image quality. Mittal et al. [20] introduced an NR general Natural Image Quality Evaluator (NIQE), which relies solely on the measurable deviation of statistical regularity in the spatial domain to estimate image quality. To investigate the impact of temporal information on image quality, Mittal et al. [21] further developed a model to compensate for Video Intrinsic Integrity and Distortion Evaluation Oracle (VIIDEO). Saad et al. [22] proposed a VQA BLIINDS-II metric, called VB-II for short, which establishes statistical and motion models for video quality evaluation. Dendi et al. [23] introduced a video quality evaluation indicator that utilizes parameterized statistical models for computing the spatio-temporal features of conventional videos. This method is briefly referred to as 3DSTGabor. However, the existing traditional IQA/VQA methods cannot effectively evaluate the quality of virtual viewpoint videos because of the different distortion characteristics. For example, the traditional 2D IQA/VQA methods underestimate the impact of local rendering distortions on image/video quality. So, it is necessary to study objective QA for synthesized virtual viewpoint videos [24].

(2) NR synthesized IQA/VQA: For NR synthesized IQA/VQA metrics, methods based on Natural Scene Statistics (NSS) are widely used. For example, Gu et al. [25] introduced a synthesized IQA metric based on the Auto-regression Plus Thresholding (APT) method. Furthermore, Gu et al. [26] extended a multiscale NSS (MNSS) method to evaluate the quality of synthesized images. Some researchers have further refined the types of synthesized distortions. For example, Tian et al. [27] put forward an NR IQA method of Synthesized Views (NIQSV), which used morphological operations on the virtual view assessment. Subsequently, they improved the NIQSV to NIQSV+ metric [28]. In addition, some scholars convert images to transform domains to highlight the distortions. For instance, Wang et al. [29] presented a synthesized IQA method, which extracted geometric, sharpness, and complexity characteristics in the transform domain. Fang et al. [30] used saliency guidance to extract local features in the spatial and log-Gabor domains, and extracted global blur features in the multiscale and multi-orientation wavelet subband domain, combined with both for synthesized image quality assessment. Recently, more scholars have focused on designing synthesized VQA methods. For example, Sandić-Stanković et al. [31] introduced an NR synthesized VQA metric based on Morphological Wavelet with Threshold (NR-MWT). Zhou et al. [32] presented an NR VQA method that relies on measuring the Flickering Distortion Intensity (FDI). To explore higher translational DoF, Jin et al. [10] proposed an NR windowed 6DoF synthesized VQA metric by measuring the 2DoF rendering distortion, 2DoF path navigation distortion, and compression distortion.

In conclusion, the majority of mainstream synthesized IQA/VQA metrics are currently limited to the 1DoF stage. As a result, there is an urgent need to develop objective VQA methods for higher-DoF synthesized videos. In response to this challenge, we propose an NR arbitrary translational 6DoF VQA metric, in which the different distortions, especially the distortions caused by viewpoint switching and virtual view rendering in arbitrary directions, can be effectively measured.

## 3. Subjective Quality Dataset of Arbitrary Translational 6DoF Video

### 3.1. Generation of Arbitrary Translational 6DoF Videos

The user’s perceived experience of the final synthesized video depends on three main technical steps. (1) The captured videos need encoding/decoding to reduce the pressure of data transmission. (2) The user’s perceived videos include both the original camera-captured video and the virtual video obtained through DIBR technology. (3) The different preferences of users when switching viewpoints will generate a variety of view switching paths in 3D space. Therefore, the distortions caused by compression, rendering artifacts, and view switching paths often deteriorate the subjective visual quality. Hence, we simulate the corresponding distortions at each stage to generate arbitrary translational 6DoF videos.

**Figure 2:**
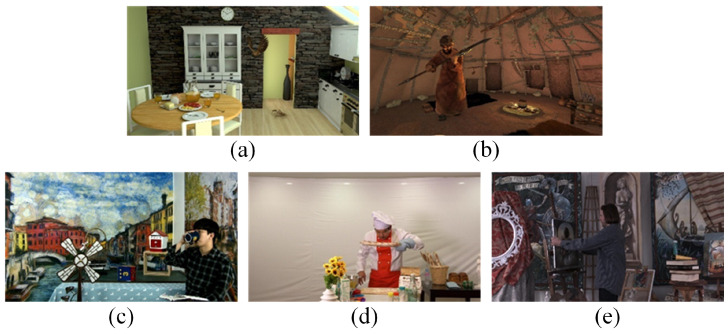
Five video sequences used in the arbitrary translational 6DoF synthesized video quality database. (**a**) *OrangeKitchen*. (**b**) *OrangeShaman*. (**c**) *ETRIBreaktime*. (**d**) *ETRIChef*. (**e**) *TechnicolorPainter*.

Five sequences presented in the form of sparse light fields are selected. They are “OrangeKitchen” [33], “OrangeShaman” [33], “ETRIBreakTime” [34], “ETRIChef” [35], and “TechnicolorPainter” [36], as shown in Figure 2. Among them, the first two are computer-generated sequences, while the last three are sequences captured using cameras, ensuring a diverse range of video content. Computer-generated sequences provide a controlled environment to systematically test the metric with specific distortions. In contrast, natural sequences, from real-world scenarios, involve more complex and diverse content, ensuring the metric can handle unpredictable distortions. By using both types of sequences, we ensure the versatility of the subjective database. This also lays the foundation for the objective metric to perform well in both theoretical and real-world scenarios.

The original video, which includes both the original texture and depth information, is compressed using the VVC VTM-14.0 reference platform [37]. Specifically, three levels of quantization parameters (QPs) are applied, each with different combinations of texture and depth QPs, namely, (35, 42), (40, 45), and (45, 48). Note that for high-resolution images, lower QP pairs result in compression distortions that are difficult to discern. Therefore, the QP range selected here is based on the subjective visual discernibility threshold for high-resolution images [13]. Additionally, the scenario of uncompressed original video is also considered, resulting in a total of four compression levels in the database.

**Figure 3:**
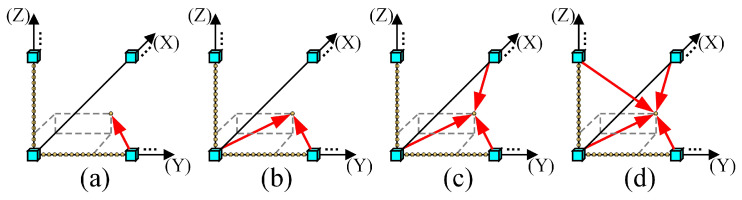
Four rendering schemes of arbitrary translational 6DoF synthesized video quality database. (**a**) S1. (**b**) S2. (**c**) S3. (**d**) S4.

The virtual viewpoints between adjacent original views are primarily generated by the combination of VSRS 4.3 [38] and the four reference view-rendering algorithm [39]. To generate arbitrary translational 6DoF video, we utilize four rendering schemes to generate virtual viewpoints. Specifically, each virtual viewpoint video is rendered by the nearest one, two, three, and four original viewpoints, respectively. For convenience, these four methods are abbreviated as S1, S2, S3, and S4, as illustrated in Figure 3a–d. It should be noted that when the number of virtual viewpoints is small, the viewer will perceive a jitter phenomenon. On the other hand, rendering too many virtual viewpoints can lead to significant computational redundancy. Considering the diversity of the selected sequence camera spacing, approximately 10–15 virtual viewpoints are rendered to avoid the interference of jitter [40]. Consequently, we set the number of virtual viewpoints between adjacent original views as 15, ensuring the consistency of all the synthesized videos.

**Figure 4:**
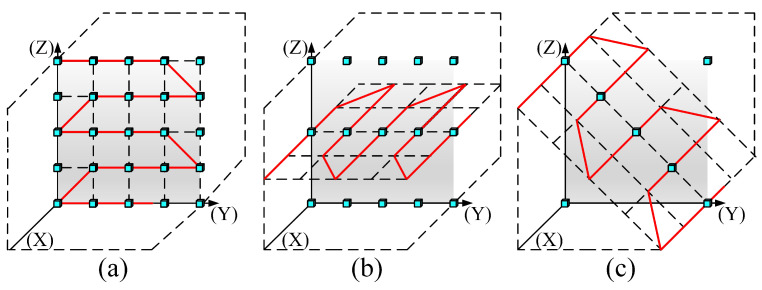
Three view switching paths of arbitrary translational 6DoF synthesized video quality database. (**a**) P1. (**b**) P2. (**c**) P3.

The flexibility of the immersive video system enables the users to freely switch viewpoints according to their preferences. We set three view switching paths to simulate users’ subjective selection behaviors. Figure 4a–c show the view switching paths in the YZ 2D plane (P1), XY 2D plane (P2), and XYZ 3D space (P3), respectively. P1 and P2 simulate restricted translational view switching, while P3 simulates arbitrary translational view switching. It should be noted that for the sake of research controllability, observers cannot switch observation paths during the testing period. And due to the subjectivity of path switching, the tested video has no reference path to rely on (i.e., there is no ideal path that everyone likes). Additional, different from the limitations of up–down and left–right view switching in the proposed windowed 6DoF database [10], this study aims to further explore the quality degradation problem of synthesized videos with arbitrary degrees of freedom after mixing forward and backward view switching. Additionally, the viewpoint navigation approach that we chose is closer to a grid layout and has the following advantages: (1) it ensures an even distribution of viewpoints in space, covering as many viewing angles as possible, thus presenting a variety of distortion effects from different perspectives; (2) it maintains equal spacing between viewpoints, making transitions between viewpoints smoother; (3) it helps evaluators more easily observe the relationships between different viewpoints, such as left–right, up–down, and front–back movements, reducing the occurrence of 3D dizziness.

In summary, the arbitrary translational 6DoF synthesized quality database comprises a total of 240 videos, including five sequences, four compression levels, four rendering schemes, and three view switching paths. The specific characteristics of the database are enumerated in Table 1.

### 3.2. Subjective Quality Testing

The subjective quality testing is conducted according to ITU-RBT.500 [14]. Since the database does not include reference videos, we select the single stimulus continuous quality grading method to assess the video scores. The ACR-HR method is utilized to achieve consistent video scores. Moreover, 30 observers (17 females and 13 males, aged between 22 and 42, with perfect vision) are summoned to participate in the subjective evaluation experiment. First, each observer needs to watch a test video with instructions to roughly understand the distortion type and degree present in the database. Then, the test videos are randomly selected and played only once. Finally, the observers are asked to rate the video quality on a five-point scale (integers from 1 to 5), where the quality and scores exhibit a positive correlation. The total frame number of each test video is controlled at 300, with a frame rate of 25FPS. It takes approximately 12 s to complete a video, and a rating of 3–5 s will be given after each ends. There are a total of five sequences in the database. After scoring three sequences, a ten-minute rest period will be arranged to alleviate visual fatigue. Overall, the scoring duration will last for 1–2 h.

Subsequently, the score set is further filtered to eliminate the interference of outliers on the video perception quality. Generally, about 95% of the confidence interval of the scores is retained [14]. Therefore, two observers (minimum and maximum scores) from 30 participants are removed. Finally, the $MOS_{q}$ of a arbitrary translational 6DoF video can be calculated as

(1) $$\begin{array}{c} {\mathrm{MOS}}_{q}=\frac{1}{K}\sum_{p=1}^{K}A_{p,q} \end{array}$$

where $A_{p,q}$ is the score assigned to the *q*th video by the *p*th observer, *K* is the number of scoring participants after excluding outliers [14], and K=28.

### 3.3. Analysis of Subjective Testing Results

**Figure 5:**
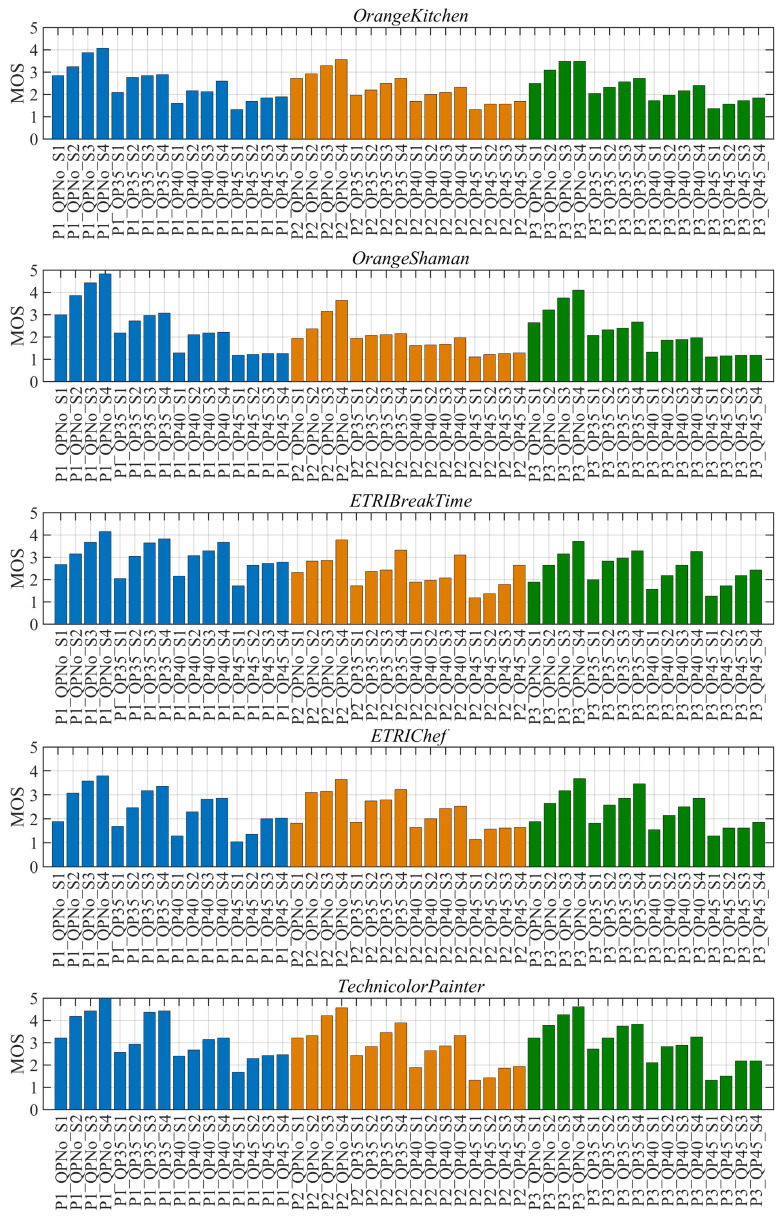
MOS distribution of arbitrary translational 6DoF videos.

Figure 5 illustrates the subjective score distribution of the arbitrary translational 6DoF videos. Using OrangeKitchen as an example, explain the meaning of each parameter. For convenience, different compression levels are represented by the QP values of texture videos. Taking the abscissa of the first column as the example for introduction, “P1_QPNo_S1” signifies that the video is generated based on view switching path P1, the original video, and its virtual viewpoint is rendered by rendering scheme S1. By maintaining consistency between the two distortion factors, the following conclusions can be drawn: (1) The quality scores of the arbitrary translational 6DoF videos are distributed across five scales, ranging from excellent to poor, and the videos can be effectively distinguished by the naked eye. (2) The MOS shows a generally increasing trend from S1 to S4, with occasional plateaus, indicating a positive correlation between the quality of the video and the number of original views used for rendering. (3) The MOS of P1 is slightly higher than P2 and P3, which means that the view switching path, including forward–backward movement, degrades the overall video quality to a certain extent. Additionally, the MOS decreases with the increase in the QPs, indicating that large QPs will cause video quality degradation. This also confirms the conventional rule in video coding.

In short, the proposed database considers not only the individual compression distortion, rendering distortion, and view switching distortion but also the uncertain combination of three distortions. The complex distortion environment significantly amplifies the difficulty of the subjective experiment. Fortunately, the experimental results of the database effectively verified the rationality of the distortion types in the simulated arbitrary translational 6DoF video, and also verified the effectiveness of the designed subjective quality testing method. Note that the proposed subjective database does not include reference information. This is because the virtual viewpoints we generate do not exist in real life. If reference information were to be included, we would need to render from an existing viewpoint (A) to another viewpoint (B), comparing the distortions at viewpoint B with the corresponding real viewpoint. However, this approach would only allow for a limited number of fixed-position virtual viewpoints, which cannot support path navigation and contradicts the original design of our database. Therefore, our database is not suitable for an objective full-reference method.

## 4. Objective VQA Metric for Arbitrary Translational 6DoF Videos

### 4.1. Distortion Analysis and Objective Metric Framework

**Figure 6:**
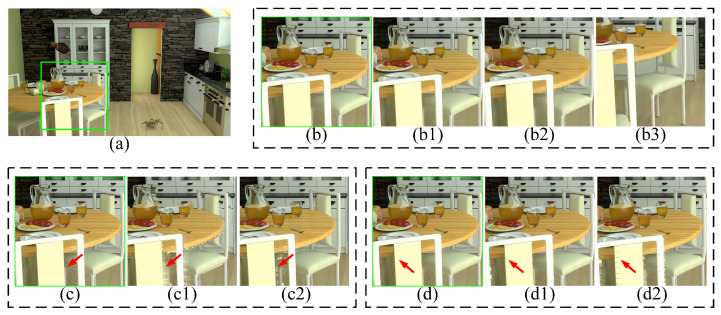
Various distortion types in the OrangeKitchen synthesized sequence. (**a**) P1_QPNo_S1_161. (**b**–**d**) are the enlargements of the green box. The (**b**) series visualizes compression distortions; the (**c**) series visualizes rendering distortions; and the (**d**) series visualizes crack distortions. Specifically, these are shown as (**b1**) P1_QP40_S1_161. (**b2**) P1_QP45_S1_161. (**b3**) P1_QP45_S1_225. (**c1**) P1_QPNo_S1_168. (**c2**) P1_QPNo_S4_168. (**d1**) P3_QPNo_S1_164. (**d2**) P3_QPNo_S1_168.

Figure 6 shows the various distortion types of the OrangeKitchen synthesized video sequence. To compare the video quality under various distortion conditions, we use four parameters to represent each synthesized video. For instance, “P1_QPNo_S1_161” indicates the 161st frame of the arbitrary translational 6DoF synthesized video, employing view switching path P1, without compression processing, and generating virtual viewpoints using rendering scheme S1. By analyzing the appearance of these distortions, the following conclusions can be drawn:

(1) Figure 6(b,b1,b2) are used to explore the impact of compression levels on the video quality. The compression distortion is globally distributed in all three video frames. As the QP increases, the frame gradually blurs. Furthermore, by comparing Figure 6(b2) with Figure 6(b3), we notice that the compression distortion remains global distribution even during video playback, specifically from the 161st frame to 225th frame. This indicates that the appearance of compression distortion exhibits temporal consistency.

(2) Figure 6(c,c1) reveal the variations of rendering distortion across different frames. The geometric rendering distortion is locally distributed in the edge area, as indicated by the red arrows. The position of rendering distortion changes from the 161st frame to 168th frame, indicating temporal inconsistency. Additionally, Figure 6(c1,c2) exhibit distortions resulting from rendering schemes S1 and S4, respectively. Clearly, S1’s distortion is more significant than S4’s.

(3) Figure 6(d,d1) are used to explore the impact of different view switching paths on the video quality. P1 has two translational DoFs in the 2D plane. Besides the two DoFs of P1, translational DoF, that is, moving forward and backward, is added.Apparently, the added translational DoF incurs a new type of distortion named “crack distortion”. The distortion is uniformly distributed throughout the video. Furthermore, by comparing Figure 6(d1) with Figure 6(d2), we find that the degree and position of the crack distortion also change during video playback. For example, the width of crack distortion at the back of the chair in Figure 6(d2) is significantly larger than that in Figure 6(d1). Therefore, the crack distortion is temporally inconsistent.

**Figure 7:**
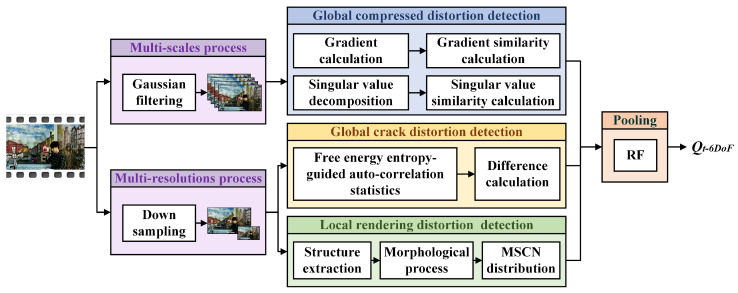
The framework of objective VQA metric for arbitrary translational 6DoF synthesized video.

Based on the above analysis, we propose an objective no-reference (NR) VQA method for arbitrary translational 6DoF videos, as illustrated in Figure 7. To address the temporal inconsistency of crack and rendering distortions, we initially downsample the video into a multi-resolution space. Next, we employ auto-correlation statistics and standard deviation calculations to detect crack distortion. Additionally, rendering distortion is identified by extracting the structure using morphological processes and counting the MSCN distribution. Furthermore, to handle the global distribution and temporal consistency of compression distortion, the video is filtered into several blur scales. The compression distortion is then evaluated using gradient and singular value similarities. Finally, a regression model is utilized to obtain the overall video quality score.

### 4.2. Multi-Resolution and Multiscale Spaces

**Figure 8:**
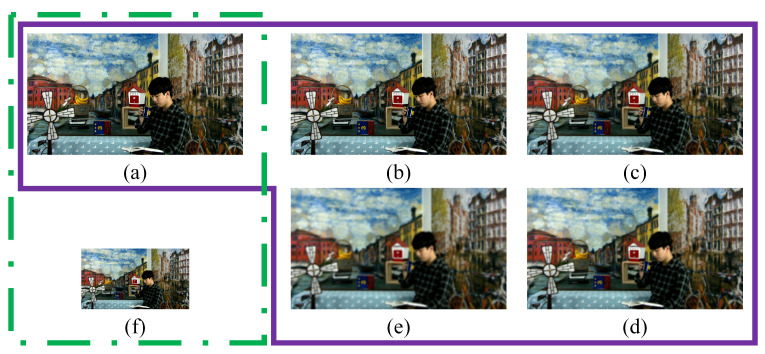
Multi-resolution and multiscale spaces of arbitrary translational 6DoF synthesized video. (**a**) Original frame. (**b**) First filtered frame. (**c**) Second filtered frame. (**d**) Third filtered frame. (**e**) Fourth filtered frame. (**f**) Double downsampling video frame.

For certain distortions with temporal inconsistency, the users’ perceived video quality can be influenced by the viewing distance. For example, the distortion area of local rendering distortion will visually shrink as the resolution decreases [26]. Similarly, the overall crack distortion, characterized by thin strip shapes, will become imperceptible while the resolution decreases. Therefore, the distortions with temporal inconsistency are greatly affected by the video resolution. Hence, to simulate the user’s perception environment, we establish the multi-resolution space, which is illustrated by the green box in Figure 8. Figure 8a represents the original resolution, while Figure 8f represents the resolution after double downsampling. It can be observed that when the resolution decreases, the eyes tend to focus more on the overall semantics and ignore the impact of subtle distortions of the video quality.

Additionally, Lowe et al. have proven that the Gaussian function is highly suitable for constructing the scale space of images and videos under various hypothetical environments [41]. In this study, we construct a multiscale space that contains the original frame and four Gaussian low-pass filtered frames. Let $I_{i}$ be $i^{th}$ low-pass filtered frame in the scale space, F be the original frame, $I_{i}$ can be obtained by convolving F with a Gaussian kernel, which can be expressed as

(2) $$\begin{array}{c} I_{i}\left(x,y\right)=F\left(x,y\right)*G_{i}\left(x,y\right) \end{array}$$

where $G_{i}\left(\cdot \right)$ is the Gaussian kernel, *x* and *y* are horizontal and vertical pixel indexes, and ∗ is the convolution operation. When *i* equals 1, 2, 3, and 4, we set the standard deviation as 2, 4, 6, 8, the corresponding Caussian kernel size as 3×3,9×9,15×15,21×21, respectively, and obtain four low-pass-filtered frames in the multiscale space.

The purple box in Figure 8 shows the multiscale space. Figure 8a represents the original video frame while Figure 8b–e correspond to scaled video frames with standard deviations of 2, 4, 6, and 8, and their corresponding Gaussian filter sizes are 3×3, 9×9, 15×15, and 21×21, respectively. Clearly, the low-filtered frames in the multiscale space are different versions of the original frame with various degrees of blur. By comparing Figure 8a with Figure 8e, the blur caused by low filtering on a high-quality original frame is significant and can be easily perceived. However, the appearance difference between Figure 8d and Figure 8e is challenging to discern. It verifies that the distortion, caused by continuous low-pass filter operation on an initially blurred frame, approaches infinity and is difficult to distinguish with the naked eye. This trend indicates that the similarity between the scaled frame and the original frame in the multiscale space can be utilized to effectively measure the degree of blur.

### 4.3. Crack Distortion Assessment

Psychophysical and neuroscience studies indicate that the human visual system has evolved to effectively interpret natural environments. The free energy entropy principle supports this by suggesting that the brain uses internal models to process visual information. Research shows that the brain reduces free energy to better align predictions with perceptions, minimizing prediction errors and system entropy, a process consistent with predictive coding [42]. The auto-correlation models can be applied to predictive coding, proving valuable for tasks such as saliency detection and Medical anatomy [43,44], highlighting the suitability of auto-correlation models for analyzing pixel-level energy variation characteristics.

**Figure 9:**
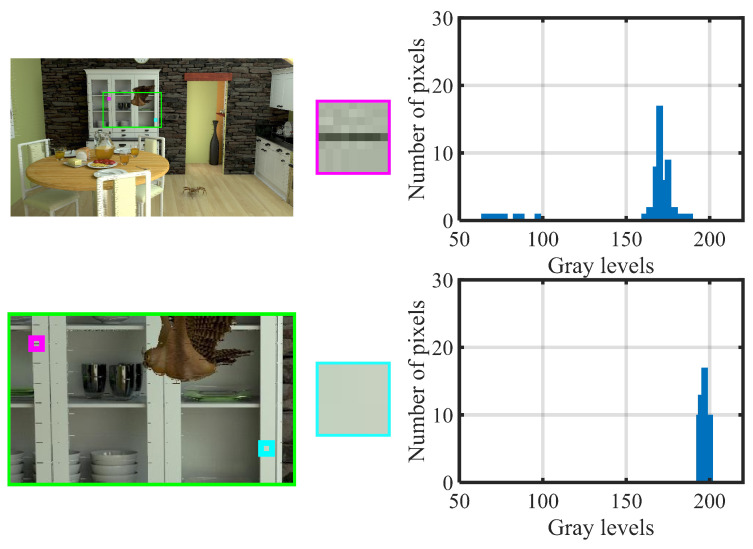
Illustration of pixel auto-correlation difference between the frames with and without crack distortion in arbitrary translational 6DoF video.

Figure 9 illustrates the pixel auto-correlation difference between frames with and without crack distortion. The left subfigures show a test video frame from the synthesized sequence OrangeKitchen, with a zoomed-in section marked by a green box. The middle subfigures present an enlarged view of this area, depicting local regions with and without crack distortion. The right subfigures display the corresponding gray histograms. It is evident that the crack-distorted regions have a wider gray-level range, indicating greater pixel variation compared to non-distorted areas, which show pixel consistency and fewer gray levels. Based on these observations and the principles mentioned above, we use pixel correlation with neighboring pixels to measure crack distortion.

Since pixels exhibit correlation with their neighboring pixels, let $V_{l}$ be a pixel at *l*, which can be constructed by

(3) $$\begin{array}{c} V_{l}=V_{\Delta (l)}\cdot \Lambda +\delta \end{array}$$

where Δ(l) is the index of neighborhood pixels of *l*, and $\Delta \left(l\right)=\left\{1,2,\ldots ,\Phi \right\}$. It is worth noting that we only consider the case where the test index locates at the central position. Hence, $\Phi =\theta^{2}-1$, where θ is an odd number starting from 3. $V_{\Delta (l)}$ is the set of neighborhood pixels. Λ is the vector to ensure that the pixel auto-correlation equation holds. Let β denote the rank of the auto-correlation vector, i.e., $\Lambda ={\left(\beta_{1},\beta_{2},\ldots ,\beta_{\Phi }\right)}^{T}$, where Φ is the dimension of the auto-correlation vector. δ represents the error between the actual pixel value and its corresponding predicted pixel value.

Furthermore, the auto-correlation vector Λ is investigated. It is known that a smaller error δ means more accurate pixel auto-correlation. Additionally, assuming that Formula (3) is satisfied, the variation in Φ within a certain range will not impact the relationship between the current pixel and its neighboring pixels. Based on this, we take another appropriate range of neighborhood pixels $\Phi_{1}$, i.e., $\theta_{1}=\theta +2$ and $\Phi_{1}=\theta_{1}^{2}-1$, to establish the pixel auto-correlation, which can be written as

(4) $$\begin{array}{c} \Lambda =\underset{\Psi }{\arg}\min\sqrt{\sum_{\Delta_{1}\left(l\right)}\sum_{\Delta (l_{1})}\left(V_{\Delta_{1}\left(l\right)}^{T}-V_{\Delta (l_{1})}\cdot \Psi \right)} \end{array}$$

where $\Delta_{1}\left(l\right)$ is the neighborhood pixels of *l*, and $\Delta_{1}\left(l\right)=\left\{1,2,\ldots ,\Phi_{1}\right\}$. $V_{\Delta_{1}\left(l\right)}^{T}$ is a set of neighborhood pixel values with a matrix dimension of $\Phi_{1}\times 1$. $\Delta (l_{1})$ is an index set of neighborhood pixels of $l_{1}$, and $l_{1}$ refers to an index which the corresponding pixel belongs to $V_{\Delta_{1}\left(l\right)}$. Therefore, the matrix dimension of $V_{\Delta (l_{1})\}}$ is $\Phi_{1}\times \Phi$, and the matrix dimension of Ψ is Φ×1. Based on the formula, a least squares method is used to obtain the optimal auto-correlation vector, which can be represented as

(5) $$\begin{array}{c} \Lambda =\frac{V_{\Delta (l_{1})}^{T}\cdot V_{\Delta_{1}\left(l\right)}^{T}}{V_{\Delta (l_{1})}^{T}\cdot V_{\Delta (l_{1})}} \end{array}$$

As is known, a small error δ means a consistent correlation between the current pixel and its neighboring pixels. Therefore, the proposed auto-correlation model is used to calculate the error between the actual pixels and the predicted pixels. Subsequently, an auto-correlation threshold *d* is introduced to differentiate cracked pixels from natural pixels. Specifically, if the error value exceeds *d*, we classify it as a crack pixel. Next, the ratio of crack pixels in the video frame is recorded as $R_{F}$. Considering that the crack varies in the temporal domain, we ultimately calculate the standard deviation of $R_{F}$ across all frames as a feature to quantify the stability of crack distortion, which can be expressed as

(6) $$\begin{array}{c} {\hat{f}}_{V}=\mathrm{STD}\left(R_{F}\left(t\right)\right),t\in \left[1,T\right] \end{array}$$

where STD represents the standard deviation function, *t* denotes the index of video frames, and *T* represents the total number of video frames.

Furthermore, users tend to be affected by the viewing distance or video resolution due to the distortion characteristics present in the inconsistent temporal domain. Therefore, the feature of crack distortion is also measured for the video after double downsampling, represented as $\overset{ˇ}{f_{V}}$. As a result, the total features of crack distortion in the arbitrary translational 6DoF synthesized video can be integrated and expressed as $f_{\mathrm{cra}}=\left[\hat{f_{V}},\overset{ˇ}{f_{V}}\right]$.

### 4.4. Rendering Distortion Assessment

Rendering distortion typically occurs in the edge area of the object and is easily perceived by the human eye. However, it is not easy for computers to detect this kind of local distortion because of the inferior scene analysis ability. Hence, the primary objective of this study is to mimic the human eye’s attention mechanism, and identify the key distribution area of rendering distortion.

When rendering distortion happens, users’ perceptual attention will be distracted. Consequently, the users pay less attention to the texture details of the video frame [45]. Hence, we extract the structure of the video frames to eliminate the interference of texture details. It is known that image filtering has been proven effective in suppressing noise and extracting structural information in various computer vision applications [46]. Thus, we first use an SD filter to extract the structure of video frames [47]. Specifically, the objective function R can be expressed as

(7) $$\begin{array}{c} R\left(O\right)=\sum_{l}e_{l}{\left(O_{l}-F_{l}\right)}^{2}+\lambda \Omega \left(O,W\right) \end{array}$$

where F is the input video frame, O is the output video frame (dynamic guidance), W is the static guidance, and $F_{l}$ and $O_{l}$ are the pixel values at *l*. The first item is the fidelity item, which coordinates the F and O, with a confidence coefficient $e_{l}$ and $e_{l}\geq 0$. The second item is the regularization item, ensuring that O remains smooth while retaining prominent features, with a regularization parameter λ.

**Figure 10:**
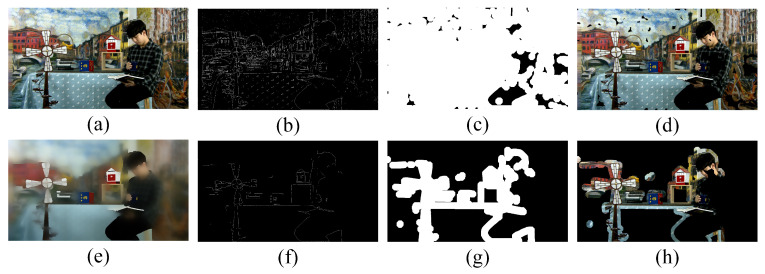
The rendering distortion extraction comparison between original image and SD-filtered image in arbitrary translational 6DoF video. (**a**) Original video frame. (**b**) Edge of (**a**). (**c**) Mask image of (**a**). (**d**) Rendering local image of (**a**). (**e**) SD-filtered image. (**f**) Edge of (**e**). (**g**) Mask image of (**e**). (**h**) Rendering local image of (**e**).

After extracting the structure, the Sobel operator is employed to obtain the edges. Subsequently, the edge image is expanded by morphological processing to create a mask image. The final target image, referred to as the rendering local image, is obtained by multiplying the mask image by the test video frame. Figure 10 shows the rendering distortion extraction comparison between the original image and SD-filtered image in an arbitrary translational 6DoF video. Figure 10a,e are the original image and the SD-filtered image. Clearly, the SD filter effectively weakens the texture detail and highlights the structural information of the video frame. Figure 10b–d show the edge, mask, and rendering local images of the original image. Figure 10f–h show the edge, mask, and rendering local images of the SD-filtered image. In Figure 10d, the extracted rendering regions are inconsistent with human perception. By contrast, in Figure 10h, the region where rendering distortion occurs has been accurately extracted, and the visual results obtained align well with subjective perception. This confirms the significance of the structure extraction operation in accurately capturing the regions affected by rendering distortion.

**Figure 11:**
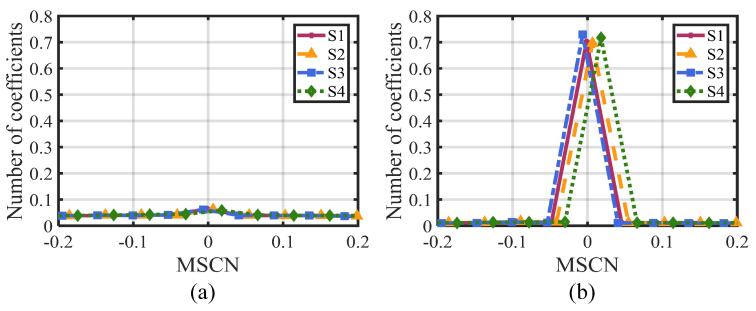
MSCN distribution of arbitrary translational 6DoF synthesized video based on different rendering schemes. (**a**) MSCN distribution of original video frame. (**b**) MSCN distribution of rendering local image.

Next, the rendering local image is normalized using the Mean Subtracted Contrast Normalized (MSCN) coefficient [48]. Figure 11 illustrates the MSCN distribution of video frames based on four different rendering schemes. Specifically, Figure 11a,b display the MSCN distribution of the original video frame and the rendering local image, respectively. By comparing these two figures, two conclusions can be drawn: (1) The MSCN distribution in the original video frame is almost indistinguishable, while the differences in the MSCN distribution are relatively obvious in the rendering local image. This proves the effectiveness of the structure extraction operation in distinguishing different distorted features. (2) In Figure 11b, the MSCN coefficients exhibit different distribution shapes and positions produced by different rendering schemes. Therefore, it can serve as an effective feature to assess the impact of rendering distortion on the video quality.

Lastly, a Gaussian mixture model (Generalized Gaussian Distribution (GGD) and Asymmetric Generalized Gaussian Distribution (AGGD)) is used to extract the multidimensional features of rendering distortion [48]. These features are abbreviated as $f_{M}$. Therefore, the rendering distortion features of video can be represented as

(8) $$\begin{array}{c} \dot{f_{V}}=\frac{1}{T}\sum_{t=1}^{T}f_{M}\left(t\right) \end{array}$$

As rendering distortion is also inconsistent in the temporal domain, the rendering distortion features are also computed for double downsampling videos, denoted as $\ddot{f_{V}}$. Therefore, the rendering distortion features of the arbitrary translational 6DoF synthesized video are summarized as $f_{ren}=\left[\dot{f_{V}},\ddot{f_{V}}\right]$.

### 4.5. Compression Distortion Assessment

Unlike rendering distortion, compression distortion globally distributes in the frame. The appearance of compression distortion is blur in terms of human visual perception. When the quantization parameter increases, the entire image becomes blurry, and the edges gradually weaken. Hence, rendering distortion changes the global structure information. Gradient information has been extensively applied in capturing the structural features [49]. Thus, we calculate the gradient similarity between each scaled frame and the original frame to assess the video quality corresponding to rendering distortion. The gradient map $B_{i}$ of each scaled frame can be calculated as

(9) $$\begin{array}{c} B_{i}=\frac{\left|I_{i}*g_{h}\right|+\left|I_{i}*g_{v}\right|}{2} \end{array}$$

where $g_{h}$ and $g_{v}$ are gradient templates in the horizontal and vertical directions for convolution, $g_{h}=\left[\begin{array}{c} -1\;0\;1 \end{array}\right]$, $g_{v}={\left[\begin{array}{c} -1\;0\;1 \end{array}\right]}^{T}$, and *i* is the frame index in multiscale space, i=0,1,2,3,4. $I_{0}$ represents the original frame, $I_{1}$, $I_{2}$, $I_{3}$, and $I_{4}$ are low-pass-filtered frames. Afterward, the similarities between the scaled gradient maps and the original gradient map are computed as

(10) $$\begin{array}{c} {\mathrm{SG}}_{j}=\frac{2B_{j}B_{0}+\varepsilon }{B_{j}^{2}+B_{0}^{2}+\varepsilon } \end{array}$$

where $B_{0}$ is the gradient map of the original frame, $B_{j}$ are the gradient maps of the filtered frames, and j=1,2,3,4, ε is a minimal constant to avoid division by zero. Thus far, the gradient similarities of a video frame $f_{F}^{\prime }$ can be expressed as

(11) $$\begin{array}{c} f_{F}^{\prime }\left(j\right)=\frac{1}{M\times N}\sum_{x=1}^{M}\sum_{y=1}^{N}{\mathrm{SG}}_{j}\left(x,y\right) \end{array}$$

where *M* and *N* are the length and width of the video frame. Ultimately, the gradient similarities of a synthesized video $f_{V}^{\prime }$ can be written as

(12) $$\begin{array}{c} f_{V}^{\prime }=\frac{1}{T}\sum_{t=1}^{T}f_{F}^{\prime }\left(j,t\right) \end{array}$$

**Figure 12:**
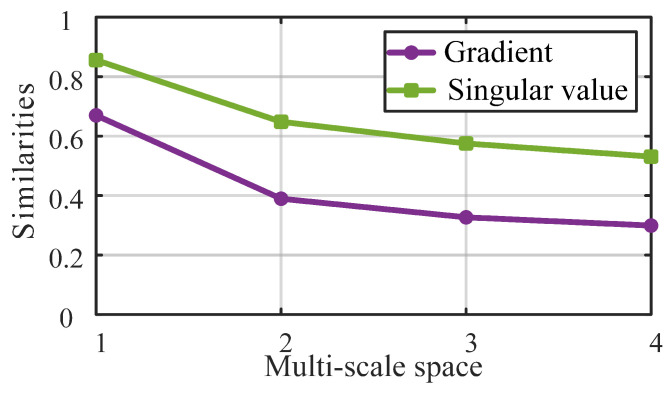
The gradient and singular value similarities between scaled video frames and the original video frame.

The purple curve in Figure 12 illustrates the gradient similarities between the scaled frames and the original frame. It can be seen that the similarities are high when the video frame is relatively clear, but as the frame is gradually blurred (with a scale index from 1 to 4), the similarities decreases, and the downward trend slows down. This phenomenon indicates that the gradient similarity reflects the sharpness of the unknown visual content in a regular manner. Thus, we use it as a feature to effectively measure the overall compression distortion.

Similarly, singular value decomposition has also been proven to be effective in measuring the internal structure changes of video due to its advantages of simplifying data and removing noise [50]. Therefore, a video frame F can be regarded as a matrix, and decomposed as

(13) $$\begin{array}{c} F={\mathrm{UCV}}^{T} \end{array}$$

where U is an M×M matrix whose columns represent the left singular vectors of F, V is an N×N matrix whose columns represent the right singular vectors of F, and C is an M×N matrix with its diagonal elements being the singular values of F. Both U and V are orthogonal matrices. If we denote α as the rank of F, then the singular value vector c can be represented as $c=(\alpha_{1},\alpha_{2},\ldots ,\alpha_{\varphi })$, where φ is the number of singular values.

Since video frames are constructed into five scale spaces, the singular values can be denoted as $\left\{c_{i},\;i=0,1,2,3,4\right\}$. Consequently, the singular value similarity between each scaled frame and the original frame $c_{0}$ can be calculated as

(14) $$\begin{array}{c} {\mathrm{SU}}_{j}=\frac{2c_{j}c_{0}+\varepsilon }{c_{j}^{2}+c_{0}^{2}+\varepsilon } \end{array}$$

where $c_{0}$ is the singular value vector of the original image, $c_{j}$ is the singular value vectors of the low-pass filtered frames, and j=1,2,3,4. Thus, the singular value similarities of a video frame can be written as

(15) $$\begin{array}{c} f_{F}^{\prime \prime }\left(j\right)=\frac{1}{M}\sum_{\varphi =1}^{M}{\mathrm{SU}}_{j}\left(\varphi \right) \end{array}$$

Finally, the singular value similarities of the arbitrary translational 6DoF video, denoted as $f_{V}^{\prime \prime }$, can be written as

(16) $$\begin{array}{c} f_{V}^{\prime \prime }=\frac{1}{T}\sum_{t=1}^{T}f_{F}^{\prime \prime }\left(j,t\right) \end{array}$$

The green curve in Figure 12 represents the singular value similarities between the scaled frames and the original frame. Similar to gradient similarity, as the blur degree of the frame intensifies, the singular value similarity decreases, and the decreasing trend gradually slows down. Clearly, the singular value similarity and the gradient similarity show the same variance trend when the compression distortion, e.g., blurring, varies. Therefore, singular value similarity is also used as a feature to measure the global compression distortion in the arbitrary translational 6DoF video.

Generally, compression distortion in arbitrary translational 6DoF video can be expressed using gradient similarity and singular value similarity as features, and it can be expressed as $f_{com}=\left[f_{V}^{\prime },f_{V}^{\prime \prime }\right]$.

### 4.6. Overall Quality Prediction

The measured distortion features, i.e., crack distortion **f**_*cra*_, rendering distortion **f**_*ren*_, and compression distortion **f**_*com*_, constitute multidimensional sets. These sets need to be pooled into a one-dimensional objective score to compare with a subjective score, so as to further test the performance of the proposed objective video QA model. Therefore, the overall objective quality score of the arbitrary translational 6DoF synthesized video, denoted as Qt−6DoF, can be expressed as

(17) $$\begin{array}{c} Q_{t-6DoF}=H\left(f_{cra},f_{ren},f_{com}\right) \end{array}$$

where H(·) is the regression function. According to the experiment, we finally use Random Forest (RF) as the regression model [51]. During the regression training phase, the features ($f_{cra},f_{ren},f_{com}$) are used for training, and the model outputs predict the quality scores. The regression error is then determined by calculating the difference between the predicted values and the labels (mos). The regression error is measured using the Mean Squared Error (MSE). Additionally, the tested subjective database is partitioned into two subsets, i.e., 80% for training and 20% for testing. The training–testing process is repeated 1000 times, and the median value is set as the final quality score.

## 5. Experimental Results and Analysis

In this section, the performance of the proposed objective VQA metric is first compared with the existing QA metrics on the proposed subjective database. Then, the impact of some parameters on the model performance is investigated. Next, the influence of the regression model and the training–testing percentage on model performance is analyzed. Finally, an ablation study is constructed to illustrate the feature contribution.

### 5.1. Performance Comparison

Four indicators, namely, the Pearson Linear Correlation Coefficient (PLCC), Spearman Rank Order Correlation Coefficient (SROCC), Kendall Rank Order Correlation Coefficient (KROCC), and Root Mean Square Error (RMSE), are employed to evaluate the performance of the objective QA metrics. Notably, the performance of the first three is higher and that of the last one is lower, indicating that the performance of the objective QA model is superior.

Table 2 presents the performance of the proposed objective metric (Tra-6DoF-S VQA) alongside existing methods on the subjective database, with the best-performing results highlighted in bold. The comparison includes a broad range of methods, such as classic and state-of-the-art traditional IQA/VQA methods (T-IQA/T-VQA), 1DoF DIBR Synthesized IQA methods (1DoF-S IQA), and windowed 6DoF Synthesized VQA methods (Win-6DoF-S VQA). It is imperative to mention that all methods are no-reference (NR) IQA/VQA models, owing to the absence of reference videos. The specific analyses are as follows.

(1) The first three items show the performance of three traditional NR IQA methods, BIQI [18], SSEQ [19], and NIQE [20]. These methods aim to evaluate the impact of common image distortion types, such as compression and noise distortions, on the image quality. Items 4 and 5 are two general traditional VQA metrics, i.e., VIIDEO [21] and VB-II [22]. They aim to evaluate the impact of general video distortion types, such as motion blur, on the video quality. According to the experimental results of these two types of QA metrics, several conclusions can be drawn. Firstly, when comparing traditional IQA and VQA methods, it is evident that the latter exhibits a higher upper limit of performance compared to the former. The rationale lies in the fact that traditional VQA methods take into account not only spatial distortion but also temporal distortion. As a result, VQA methods possess a more comprehensive ability to assess video quality. This phenomenon also verifies that the human eyes are more sensitive to dynamic distortions. Secondly, the traditional IQA/VQA metrics predominantly assess the degradation of image/video quality stemming from camera capture or environmental conditions. Many of these types of distortions are uniformly spread throughout visual content. Consequently, the overall performance of these methods on the proposed subjective database, which encompasses both global and local distortions, is not flawless. Even the PLCC of VB-II, which is the best in the first five items, is lower than that of the proposed objective VQA method. In short, the traditional IQA/VQA methods exhibit restricted adaptability when it comes to quantifying specific rendering distortions within synthesized videos. This outcome underscores the necessity of constructing a VQA metric tailored for synthesized videos.

(2) Items 6 to 10, APT [25], MNSS [26], NIQSV [27], NIQSV+ [28], and Wang [29], are five IQA metrics designed for 1DoF DIBR synthesized images. The experimental results indicate that these IQA methods perform poorly. The lowest PLCC is 0.2769, for the NIQSV method, while the highest PLCC is 0.5325, for the MNSS method. The reason for this is that these methods are designed to detect old-fashioned rendering distortion, like holes and stretching. However, as DIBR technology has matured, these distortions have been phased out from recent virtual viewpoint databases. Additionally, these methods rarely account for distortions beyond local rendering, undermining their robustness. Unfortunately, in cases where rendering distortion appears in a video, and its shape and position dynamically shift, the performance of these IQA methods further degrades. Hence, it is inefficient to employ a 1DoF DIBR synthesized IQA method to evaluate the quality of arbitrary translational 6DoF synthesized videos.

(3) Items 11 and 12 are VQA metrics designed for high-DoF synthesized video, including the windowed 6DoF synthesized VQA method [10] and the proposed method. Both methods share the characteristic of consideration of global and local distortions within a higher-DoF multimedia context. The average PLCC of the two reaches 0.8616. Therefore, the performance of these two methods is better than the traditional IQA/VQA and 1DoF DIBR synthesized IQA methods. Since the windowed 6DoF VQA method does not take into account the crack distortion caused by forward/backward view switching, its performance is slightly inferior to that of the proposed method. To sum up, the proposed objective NR VQA metric reaches the best performance in evaluating the quality of arbitrary translational 6DoF videos.

### 5.2. Performance Dependency of Multiscale Space

Explorative experiments are performed to examine the impact of the scale number of the multiscale space on the model performance. Specifically, we set the scale number of the multiscale space as 2, 3, 4, 5, and 6, and then obtained the PLCC, SROCC, KROCC, and RMSE, respectively, of the corresponding objective QA model. For example, if the scale number is set as 3, the multiscale space contains the original frame and two Gaussian low-pass-filtered frames by Formula (2) with i=1,2. The outcomes of these experiments are listed in Table 3.

(1) For scale numbers of 2, 3, and 4, the performance of the objective VQA model is competitive, but there is obviously room for improvement. This is primarily attributed to the reduction of the scale number, which directly translates to a diminished feature dimension for gradient similarity and singular value similarity. Evidently, feature extraction across a broader range of multiscale space can encompass a greater extent of distortion details, consequently yielding a more effective assessment of compression distortion.

(2) As the video scale space increases, the performance of the objective VQA model steadily converges. Specifically, when the scale number reaches 5, the model performance achieves a high performance, with a PLCC of 0.8923, SROCC of 0.8700, KROCC of 0.6967, and RMSE of 0.3886. When the scale number rises up to 6, the performance of the VQA model slightly decreases in terms of the SROCC, KROCC, and RMSE. In addition, the further increments in the scale number will increase the computational complexity. Hence, we set the scale number of the multiscale space in the VQA model as 5, according to the overall performance.

### 5.3. Performance Dependency of Neighborhood Pixel Range

Table 4 presents the performance impact of the neighborhood pixel range of the pixel auto-correlation model on the proposed VQA metric. The neighborhood pixel range is determined by the parameter θ. For instance, θ=3 means that the total number of the neighbor pixels is $3^{2}-1$. Here, we investigate the model performance when θ is set to 3, 5, 7, and 9. Additionally, the average computational time of each frame is presented. It can be seen that, with the increase in θ, the PLCC, SROCC, and KROCC slightly fluctuate. Simultaneously, the computational time of the model also experiences a substantial increase. It is worth noting that, although the model performance improves when the θ is 5 or 7, the difference is not significant compared to when the θ is 3. However, the computation time surges by 3.7 min, leading to a noteworthy efficiency reduction in the VQA model. Therefore, the parameter θ in the proposed arbitrary translational 6DoF synthesized VQA metric is ultimately set as 3.

### 5.4. Performance Dependency of Auto-Correlation Threshold

In the proposed VQA metric, the auto-correlation threshold *d* is mainly used to distinguish the crack pixels and natural pixels in the arbitrary translational 6DoF video. We investigate the impact of *d* on the performance of the proposed VQA metric. *d* is set to 20, 40, 60, 80, 100, 120, and 140, respectively, and the performance of its corresponding objective VQA model is estimated. Table 5 lists the experimental results.

(1) The performance of the proposed objective VQA model does not fluctuate significantly even when the threshold *d* changes within a large range. For example, when the threshold value *d* is 140, the model performance is the worst, i.e., the PLCC is 0.8411, which is still better than most classical and state-of-the-art objective QA metrics.

(2) While the overall performance shows little variation across *d* ranging from 20 to 140, it still improves when *d* is set between 80 and 120. Specifically, the PLCC achieves the maximum value of 0.9021 at *d* of 80. The SROCC and KROCC both achieve their maximum values at a *d* of 100. RMSE reaches its minimum value at a *d* of 120. Therefore, we ultimately set a *d* of 100 in the proposed objective VQA metric.

### 5.5. Performance Dependency of Regression Model and Training-Testing Ratio

In the proposed VQA metric, the regression model aims to map multidimensional features to a one-dimensional objective score. The ratio of training and testing refers to the proportion of samples allocated to the regression model. Both the regression model and the training–testing ratio affect the performance of the proposed VQA metric. Hence, we perform experiments to obtain the reasonable combination of regression model and training–testing ratio and achieve optimal performance. The experimental results are listed in Table 6.

Two regression models, Support Vector Regression (SVR) and RF regression, are compared. As for the performance under the same training–testing ratio, although the objective VQA model based on SVR is good, it is slightly inferior to the model utilizing RF regression. For example, when the training–testing ratio is 80-20%, the PLCC of the SVR-based objective VQA model is 0.8884. In comparison, the RF regression-based objective VQA model achieves a PLCC of 0.8923. Therefore, the proposed VQA metric adopts RF regression to generate the final quality score.

Furthermore, we also verify the impact of the training−testing ratio on the performance of the objective VQA metric. Five training–testing ratios, 50-50%, 60-40%, 70-30%, 80-20%, and 90-10%, are investigated. According to the experimental results in Table 6, two conclusions can be drawn: (1) With the increase in video training samples, the performance of the objective VQA model consistently improves. Notably, the model performs well even when the training sample is only 50%, achieving a PLCC of 0.8398. This demonstrates that the model can achieve competitive results even with limited training samples, further emphasizing the practical robustness of the proposed objective VQA metric. (2) With a training sample of 90%, the model performance is more excellent, with a PLCC of 0.9180. This outcome strongly underscores the superiority of the proposed objective VQA metric in comparison with the existing methods. It also means that the proposed metric can effectively estimate the quality of the arbitrary translational 6DoF videos.

### 5.6. Ablation Studies

Three types of distortions, compression distortion $f_{\mathrm{com}}$, crack distortion $f_{\mathrm{cra}}$, and rendering distortion $f_{\mathrm{ren}}$, extracted from the proposed objective VQA metric are utilized to construct distinct models. This approach aims to evaluate the individual impacts of these distortion assessments on the overall model performance. The experimental results, summarized in Table 7, yield three key conclusions.

(1) The single-component VQA models, Model 1 and Model 2, measure compression distortion and rendering distortion separately. They can obtain high performance results, with PLCCs of 0.8172 and 0.8563, respectively. However, Model 3 separately estimates the crack distortion, and its performance lags behind the former two models, with a PLCC of 0.6028. The reason for this is that the quality of the arbitrary translational 6DoF video is typically influenced by multiple distortions. Both the global compression distortion and local rendering distortion play significant roles during video playback, operating within the spatial and temporal domains of the video. Consequently, due to visual masking effects, observers tend to prioritize the impact of limited yet significant distortions, often disregarding the influence of crack distortion on the video quality. This ultimately contributes to the comparatively lower performance of the VQA model corresponding to crack distortion.

(2) Model 4, Model 5, and Model 6 are objective VQA models obtained by the permutation and combination of two distortion components. Clearly, the performance of the VQA models based on two distortion components is higher than that of the models with a single distortion component. Specifically, the PLCCs of Model 4, Model 5, and Model 6 are 0.8737, 0.8674, and 0.8600 respectively. Therefore, it is reasonable to consider the characteristics and distribution of various distortions and add them to the VQA model. In addition, although the performance of the single-component VQA model with crack distortion might be inferior, the ultimate model’s efficiency still benefits from the diversity introduced by crack distortion.

(3) As the number of distortion components increases, the performance of the objective VQA model improves. Ultimately, when the three distortion components are integrated, as seen in Model 7, the model performance reaches its optimal value, with a PLCC of 0.8923. The experimental result validates the contribution of features derived from compression distortion, rendering distortion, and crack distortion to the objective VQA model. Each of these components plays a pivotal role in shaping the final objective arbitrary translational 6DoF VQA metric.

### 5.7. Performance of Individual Video Sequence

Table 8 presents the RMSE performance for each sequence, which measures the model’s accuracy by calculating the difference between the predicted and actual scores. A lower RMSE indicates a better match between the prediction and true label, reflecting a better performance.

From the RMSE differences, ETRIBreaktime performs the best with the smallest RMSE, indicating the most accurate quality prediction. In contrast, TechnicolorPainter has the highest RMSE, suggesting poorer feature adaptation in the model. Visually, ETRIBreaktime shows more noticeable distortions, such as crack, rendering, and compression distortions, with clear pixel-level irregularities. This could be due to its bright colors and distinct foreground–background separation, which helps the model detect distortions. On the other hand, TechnicolorPainter has a darker, more complex scene, where distortions are harder to detect, likely contributing to the higher RMSE. This also confirms that the proposed model struggles with darker or more complex scenes, which is an area for future research and improvement.

## 6. Conclusions

In this paper, we present an exploratory approach to subjective and objective VQA metrics for arbitrary translational 6DoF synthesized videos, aimed at advancing immersive video system development. Specifically, we establish a new video quality database that emphasizes 3D spatial path navigation, examining its impact on video quality under distortions such as crack, rendering, and compression, which were previously limited to planar navigation in earlier studies. Leveraging the spatio-temporal characteristics of these distortions, we propose a no-reference objective VQA method that extracts spatial and temporal auto-correlation features and regresses them into a final quality score. The experimental results show that the proposed objective method outperforms existing approaches on the newly established subjective database.

In the future, we plan to explore methods for higher-DoF video quality assessment, focusing on incorporating more advanced statistical approaches and unsupervised deep learning techniques. By combining the high theoretical orientation of statistical analysis with the automatic feature extraction capabilities of deep learning, we aim to capture the complex distortion characteristics in videos more comprehensively.

## Figures and Tables

**Table 1:** The specific characteristics of the arbitrary translational 6DoF synthesized video quality database.

Sequences	Camera Array	Camera Space (cm)	Resolution	QP Pairs	DIBR Schemes	Paths	Syn-Video	FPS
OrangeKitchen	5 × 5	11.5 × 11.5	1920 × 1080	No, (35,42), (40,45), (45,48)	S1, S2, S3, S4	P1, P2, P3	48	25
OrangeShaman	5 × 5	10 × 10	1920 × 1080	No, (35,42), (40,45), (45,48)	S1, S2, S3, S4	P1, P2, P3	48	25
ETRIBreakTime	3 × 5	15 × 15	1920 × 1080	No, (35,42), (40,45), (45,48)	S1, S2, S3, S4	P1, P2, P3	48	25
ETRIChef	5 × 5	10 × 10	1920 × 1080	No, (35,42), (40,45), (45,48)	S1, S2, S3, S4	P1, P2, P3	48	25
TechnicolorPainter	4 × 4	7 × 7	2048 × 1088	No, (35,42), (40,45), (45,48)	S1, S2, S3, S4	P1, P2, P3	48	25

**Table 2:** Performance comparison of the proposed objective method with existing no-reference (NR) IQA/VQA objective evaluation metrics on the proposed subjective database.

Metric	Type	PLCC	SROCC	KROCC	RMSE
BIQI [18]	T-IQA NR	0.2762	0.2421	0.1666	0.8197
SSEQ [19]	T-IQA NR	0.3486	0.3237	0.2257	0.7980
NIQE [20]	T-IQA NR	0.6302	0.6087	0.4366	0.6598
VIIDEO [21]	T-VQA NR	0.3633	0.3389	0.2372	0.7972
VB-II [22]	T-VQA NR	0.8681	0.8622	0.6762	0.4299
APT [25]	1DoF-S IQA NR	0.4328	0.4053	0.2859	0.7684
MNSS [26]	1DoF-S IQA NR	0.5325	0.5227	0.3709	0.7152
NIQSV [27]	1DoF-S IQA NR	0.2769	0.2459	0.1684	0.8205
NIQSV+ [28]	1DoF-S IQA NR	0.5251	0.5267	0.3716	0.7208
Wang et al. [29]	1DoF-S IQA NR	0.5005	0.4763	0.3344	0.7363
Jin et al. [10]	Win-6DoF-S VQA NR	0.8308	0.8150	0.6364	0.4260
Proposed	Tra-6DoF-S VQA NR	**0.8923**	**0.8700**	**0.6967**	**0.3886**

**Table 3:** Performance of the proposed objective VQA metric with different scale numbers.

Scale Number	2	3	4	5	6
PLCC	0.8853	0.8892	0.8904	0.8923	**0.8936**
SROCC	0.8534	0.8630	0.8688	**0.8700**	0.8687
KROCC	0.6709	0.6816	0.6942	**0.6967**	0.6963
RMSE	0.4094	0.3977	0.3905	**0.3886**	0.3891

**Table 4:** Performance of the proposed objective VQA metric with different θ.

θ	3	5	7	9
PLCC	0.8923	0.8990	0.8946	0.8738
SROCC	0.8700	0.8703	0.8721	0.8580
KROCC	0.6967	0.7028	0.6979	0.6327
RMSE	0.3886	0.3805	0.3855	0.3953
Time (m)	2.9	5.2	6.6	7.1

**Table 5:** Performance of the proposed objective VQA metric with different auto-correlation thresholds.

*d*	20	40	60	80	100	120	140
PLCC	0.8841	0.9003	0.8905	**0.9021**	0.8923	0.8627	0.8411
SROCC	0.8258	0.8528	0.8476	0.8683	**0.8700**	0.8549	0.8269
KROCC	0.6266	0.6544	0.6557	0.6846	**0.6967**	0.6813	0.6373
RMSE	0.4487	0.4094	0.4026	0.3933	0.3886	**0.3878**	0.4418

**Table 6:** Performance of the proposed objective VQA metric with different combinations of regression models and training–testing ratios.

Regression Model	Training-Testing Ratio	PLCC	SROCC	KROCC	RMSE
SVR	50-50%	0.8293	0.8177	0.6322	0.4468
	60-40%	0.8576	0.8381	0.6430	0.4243
	70-30%	0.8750	0.8579	0.6665	0.4023
	80-20%	0.8884	0.8695	0.6788	0.3961
	90-10%	0.8980	0.8836	0.6898	0.3754
RF	50-50%	0.8398	0.8340	0.6399	0.4357
	60-40%	0.8585	0.8472	0.6545	0.4169
	70-30%	0.8759	0.8654	0.6771	0.3988
	80-20%	0.8923	0.8700	0.6967	0.3886
	90-10%	0.9180	0.8985	0.7398	0.3294

**Table 7:** Contribution of different distortion components to the proposed objective arbitrary translational 6DoF VQA metric.

Model	Distortion Component			PLCC	SROCC	KROCC	RMSE
	f_*com*_	f_*ren*_	f_*cra*_				
1	√			0.8172	0.8106	0.6251	0.4743
2		√		0.8563	0.8276	0.6366	0.4395
3			√	0.6028	0.5696	0.4630	0.6821
4	√	√		0.8737	0.8518	0.6697	0.3700
5		√	√	0.8674	0.8270	0.6444	0.4529
6	√		√	0.8600	0.8399	0.6708	0.4310
7	√	√	√	**0.8923**	**0.8700**	**0.6967**	**0.3886**

**Table 8:** RMSE of individual video sequences.

Sequences	RMSE
OrangeKitchen	0.2513
OrangeShaman	0.2929
ETRIBreaktime	0.1570
ETRIChef	0.2934
TechnicolorPainter	0.3305

## Data Availability

The data presented in this study are available on request from the corresponding author.
